# Speciation of chromium in waters using dispersive micro-solid phase extraction with magnetic ferrite and graphite furnace atomic absorption spectrometry

**DOI:** 10.1038/s41598-020-62212-7

**Published:** 2020-03-24

**Authors:** Ignacio López-García, Juan José Marín-Hernández, Manuel Hernández-Córdoba

**Affiliations:** Department of Analytical Chemistry, Faculty of Chemistry, Regional Campus of International Excellence “Campus Mare Nostrum”, University of Murcia, E-30100 Murcia, Spain

**Keywords:** Environmental chemistry, Hydrology

## Abstract

The combination of a solid-phase microextraction process with graphite furnace atomic absorption spectrometry provides a very sensitive determination method for determining chromium in waters. Freshly prepared ferrite particles are used to retain the chromium species, and then separated by a magnet without the need for a centrifugation step. The solid phase is suspended in water and directly introduced into the graphite furnace to obtain the analytical signal. The complexation of Cr(III) with ethylenediaminetetraacetate allows the selective retention of Cr(VI), and thus the speciation of the metal. The procedure is sensitive (0.01 µg L^−1^ detection limit when using a 10 mL sample aliquot) and reproducible (5% relative standard deviation for five consecutive experiments at the 0.3 µg L^−1^ level). The reliability of the procedure is verified by analysing five certified water samples.

## Introduction

The preparation of the sample in environmental analysis is a critical stage since it largely determines the quality of the results obtained and, consequently, methodologies are required that without losing efficiency and reliability be fast, affordable and sustainable^1^. The elemental trace analysis deals with the determination of metals, metalloids and non-metals that sometimes are present at very low concentrations. In biological or environmental samples, the determination is difficult because the levels of some elements may be even below the determination limit attainable in most conventional atomic techniques^2,3^. To this circumstance must be added the importance that elemental speciation has reached in recent years^4,5^, which has resulted in the development of metalomics^6^.

Within the different stages of sample preparation, the transfer of the analyte from a donor phase to another immiscible one (the acceptor phase) fulfil a double purpose since in addition the clean-up effect that avoids possible difficulties in the subsequent determination, allows a preconcentration of the analyte that facilitates measurement. The process should be carried out using simple, easily available reagents compatible with the analytical technique used for the final measurement and avoiding or minimizing the production of contaminated wastes. In most cases, the donor phase is already in a liquid state^7^ and the cleaning and separation stages are combined in a single stage. The acceptor phase may be a liquid immiscible with the donor phase, a supported or dispersed solid or a micellar phase^8^. The first two approaches are the most used in a large number of ways with their advantages and disadvantages. Liquid phase microextraction^9–11^ and the use of nanomaterials as the acceptor phase, especially when dispersed (dispersive solid phase microextraction, DSPME)^12^ have proven to be particularly useful for the purpose. The advantages of using nanomaterials in DSPME have aroused a great interest in recent years^13,14^. Due to its small particle size, the transfer of the analyte is rapid and, after the separation of the donor phase, the back-extraction of the analyte is carried out in a microvolume, that is then submitted to measurement in an instrument appropriate to deal with small volumes. When the adsorbent material has magnetic characteristics^15^ the separation of phases can be achieved by applying a magnetic field, which speeds up the overall process.

In the case of metallic species which are present at low concentrations, the final measurement stage is usually carried out using atomic absorption spectrometry (AAS), inductively coupled atomic emission spectrometry (ICP-AES) or inductively coupled plasma mass spectrometry (ICP-MS). When dealing with the extremely low concentrations of some toxic or hazardous metals in waters, ICP-MS is the best alternative since it allows very sensitive determinations of a large number of analytes. The supremacy of ICP-MS is undeniable, but this analytical technique is expensive both in terms of acquisition of the instruments and their maintenance, which sometimes put it beyond the reach of small or medium-sized laboratories. By contrast, AAS is a well-established technique in most laboratories; it is relatively cheap, consumes small amounts of gases and maintenance costs are low. The sensitivity attainable by AAS is good but below that possible with ICP-MS. However, the above mentioned modern microextraction techniques offer a way of boosting the analytical performance of AAS-based procedures by increasing sensitivity. As indicated, this methodology means the analytes can be transferred from a relatively large volume of sample to a few microlitres of extract, thus resulting in a preconcentration of the metal to be measured. In addition to liquid-liquid microextraction approaches^11,16–20^ another interesting alternative is to use solid phase extraction or, better still, micro-solid phase extraction with an appropriate solid phase followed by releasing the analyte using a suitable reagent^12,20–23^. Such a possibility is especially useful when combined with graphite furnace atomic absorption spectrometry (GFAAS) since, when using this atomization mode, only a small volume (10–20 µL) is required for the measurement. The interest of the approach further increases if, instead of separating the micro-solid phase from the liquid phase by a prolonged centrifugation step, a magnetic material is used as the solid phase since this obviates the need for centrifugation, and the application of a magnet allows the simple and rapid separation of phases^24–28^.

Ferrite particles are suitable for the above purpose since they have excellent adsorptive characteristics, and their magnetic properties enable easy separation by a magnet. This methodology has been used for the separation of a number of metallic species^29–31^ including the difficult case of chromium in waters^32–35^ but, to the best of our knowledge, in all the analytical procedures reported to date, the ferrite particles are functionalized or mixed with other solid-phases to obtain nanocomposites with magnetic properties. Full benefit is not taken of the good adsorptive properties of the ferrite particles, which are merely used as a support to render the magnetic separation feasible. Recent experiments in our laboratory^29,36^ have demonstrated that freshly prepared ferrite particles are particularly effective for retaining small amounts of species, such as arsenic and silver, which can then be measured by GFAAS resulting in analytical determination procedures with a degree of sensitivity similar to that of ICP-MS. This manuscript reports the results obtained when using this approach (freshly prepared ferrite particles for micro-solid separation followed by GFAAS measurement) for the difficult case of determining low concentrations of chromium in waters. At the best of our knowledge, there are no previous reports using non-functionalized ferrite particles for the purpose. The procedure here studied is reliable, involves a non-expensive solid reagent which is easily synthesized and allows the two forms of chromium, trivalent and hexavalent, to be discriminated, which is of interest because of their different toxicity.

## Methods

### Chemicals

Chromium (VI) and chromium (III) stock solutions (1 g L^−1^) were prepared from K_2_Cr_2_O_7_ and Cr(NO_3_)_3_.9H_2_O (Fluka, Buchs SG,Switzerland), respectively, and diluted daily to obtain suitable standard working solutions. A 0.2 M Fe(II) solution was prepared from FeCl_2_.4H_2_O and a 0.1 M Fe(III) solution was prepared from FeCl_3_.6H_2_O, the solid reagents being provided by Sigma (St. Louis, MO, EE.UU.). Despite the high purity of these chemicals, and due to the extreme sensitivity of the analytical procedure, these solutions had to be purified to remove chromium traces that would have led to excessively high blank assays. Therefore, the Fe(III) solution was prepared in a 9 M hydrochloric acid medium and a 5 mL aliquot was shaken with the same volume of n-octanol. After centrifuging and discarding the aqueous phase, the extraction was repeated with a new aliquot of the organic solvent; the two organic extracts were mixed and then iron was back-extracted twice with 2.5 mL water. In this way, most of the chromium traces initially present were removed. In the case of the Fe(II) solution, purification was carried out by passing it through a minicolumn containing an anionic exchange resin (IRA-743), that retained most of the chromium while the Fe(II) concentration remained unchanged. Other chemicals used were obtained from Merck (Darmstadt, Germany).

### Instrumentation

A Perkin-Elmer model 800 (Shelton, MA, USA) spectrometer was used for all the atomic absorption measurements. The spectrometer was equipped with a transversely heated electrothermal atomizer and a Zeeman-based correction device. The graphite atomizers as well as an automatic sampler were also obtained from Perkin-Elmer. The instrumental parameters and the heating program used are summarized in Table 1.

**Table 1:** Instrumental parameters and heating program.

Parameter			
Lamp current, mA		30	
Wavelenght, nm		357.9	
Slit, nm		0.7	
Atomizer		Transverse with L’Vov platform	
Injected sample volume, µL		20	
Chemical modifier		none	
Sample volumen, mL		10	
**Heating program**			
**Step**	**Temperature, °C**	**Ramp, s**	**Hold, s**
1: Dry	110	10	20
2: Dry	130	15	30
3: Ashing	1500	10	20
4^a^: Atomization	2500	0	5
5: Cleaning	2550	1	3

^a^Argon flow 250 mL min^−1^ in all steps, except during atomization, where the gas flow was stopped.

The permanent magnet blocks (50 × 15 × 15 mm and 86 grams weight with a strength of 33 kg) composed of Nd-Fe-B that were used to carry out the magnetic separations were supplied by Supermagnete (Gottmadingen, Germany). A common ultrasonic bath and a vortex device were also used.

### Samples and analytical procedure

#### Water samples

Six water samples were analyzed. Two of them were bottled mineral waters purchased in a local supermarket. A tap water sample was taken in the laboratory. Samples were also obtained from a natural spring and from the Segura river, which flows through Murcia, south-eastern Spain. A seawater sample was taken from a coastal marine lagoon in the same geographical area. All these samples were filtered and kept at 4 °C in plastic containers until the analyses were carried out.

#### Reference materials

In addition, five standard samples with a certified chromium content were used to verify the reliability of the results. These reference materials, namely SRM 1640a, NASS-6, SRM TM-23.4, SRM TM-25.4, and TMRain-04, were from the National Institute of Standards and Technology, the Research Council of Canada and Environment Canada.

#### Analytical procedures

The MNPs were obtained *in situ* as described elsewhere^36^, but the procedure is summarized here to help the reader. To 10 mL pure water, 0.1 mL of the 0.2 M Fe(II) solution and 0.1 mL of the 0.1 M Fe(III) solution were added. After heating at 60 °C, a small volume (40 µL) of concentrated ammonia solution was incorporated, and the mixture was submitted to ultrasounds for 4 min. The solid material was separated using a magnet and washed twice with 1 mL pure water. The MNPs thus obtained and remaining inside the tube were used directly. To determine the chromium total content, 10 mL of sample (0.3 M sodium hydrogen carbonate was incorporated to bring the pH close to neutrality, if necessary) was added to the tube containing the freshly prepared MNPs and, after shaking for a few seconds, the magnetic material was separated by applying a magnet to the external part of the tube. The supernatant was discarded, and the residue was washed twice with 1 mL water again using the magnet for the separation. Finally, a suspension was obtained by adding 0.1 mL water and homogenizing with the help of a vortex. A 20 µL aliquot was taken and introduced into the graphite atomizer before applying the heating program given in Table 1. The analytical signal (area obtained during the atomization stage) corresponded to the total chromium content. The measurement was always obtained in duplicate.

To calculate the Cr(VI) content, 0.3 mL of 1 M sodium hydrogen carbonate adjusted to pH = 7 and 0.1 mL of 0.01 M EDTA were incorporated in the sample, and the solution was heated at 60 °C for 15 min to achieve Cr(III) complexation. Next, the procedure described was repeated with another tube containing freshly prepared MNPs. The analytical signal finally obtained in the GFAAS instrument corresponded to Cr(VI). The concentration of Cr(III) was obtained by difference.

## Results

### Retention of chromium species by ferrite particles

**Figure 1:**
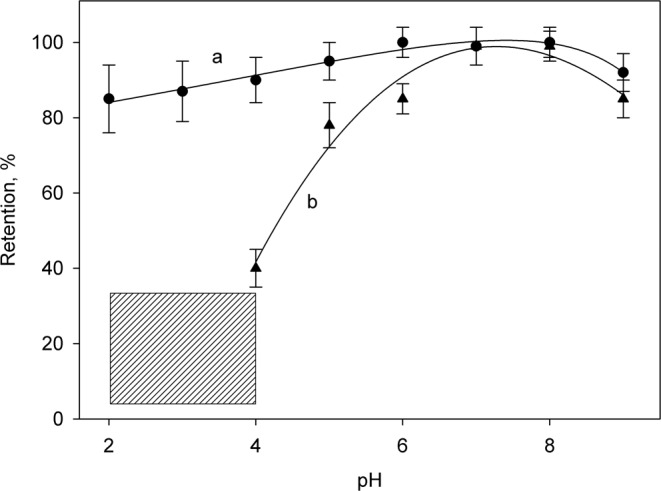
Effect of pH on the retention of Cr (VI) and Cr (III) (curves a and b, respectively) using freshly synthesized ferrite. The shaded pH zone corresponds to a partial solubilization of the ferrite particles, and so the data have a greater variability.

All the experiments were carried out using freshly prepared ferrite particles for the preconcentration step. As was to be expected, the retention of chromium species by the solid particles strongly depended on the acidity of the medium. To study this parameter, a number of solutions containing 25 µg/mL Cr(III) or Cr(VI) were treated with the magnetic material and, after separating the solid by means of a magnet, the concentration of the metal remaining in the supernatant was measured. The results shown in Fig. 1 demonstrated that the retention of Cr(VI) was high in all the pH range studied, and that the trivalent form was practically totally retained for solutions close to neutrality, but less so as the acidity was increased to pH 4. At pHs below this value the solid phase was partially dissolved. This behavior agrees with the z-potential of the ferrite particles, as reported elsewhere^36^.

### Speciation of chromium

A large number of experiments were devoted to developing a strategy that allowed trivalent and hexavalent chromium to be discriminated, i.e., to achieve a reliable chromium speciation. Since both species are retained by the MNPs at pH values close to neutrality, several complexing agents for Cr(III) were assayed in the search for a robust complex that could avoid its retention. Excellent results were found when ethylenediaminetraacetate (EDTA) was used for the purpose. This chemical forms a very stable chelate with Cr(III) although the rate of formation is slow and requires mild heating and/or the presence of an auxiliary anion (carbonate) acting as a catalyst^37^. Once it was verified that the EDTA-Cr(III) complex was not retained on the MNPs at pH values close to 7, experiments were carried out to verify that the possible speciation would be reliable. To this effect it should be noted that there is a risk that Cr(VI) may oxidize EDTA, jeopardizing a correct speciation. However, in the case here considered it was experimentally verified that such a red-ox process did not take place because the pH was not acid and the solution was heated only gently. Both the temperature and time of heating as well as the EDTA concentration were optimized and found to be 60 °C during 15 min in the presence of 1 mM EDTA. The concentration of the carbonate incorporated in the solution to achieve a pH close to neutrality and to increase the rate of formation of the EDTA-Cr(III) complex was also optimized experimentally and a 0.03 M concentration was finally selected.

To summarize, chromium speciation can be achieved by means of two consecutive experiments, as detailed in the Experimental section. The first one allows the total concentration (Cr(VI) + Cr(III)) present in the sample to be calculated. The experiment is then repeated using another aliquot of sample but in the presence of 1 mM EDTA so that only Cr(VI) is retained by the MNPs, and then obtaining again the analytical signal. The concentration of the trivalent species is obtained by difference. The reliability of the strategy was checked by preparing a set of six solutions in which the Cr(VI)/Cr(III) ratio was varied from 50 to 0.02 using a concentration level for total chromium close to 5 µg/L The recoveries of the metal for five consecutive experiments, for each of the six solutions prepared were in the 98–102% range.

### Calibration. Analytical figures of merit

Using 10 mL-sample aliquots as described in the Experimental section, calibration graphs were obtained by least-squares linear regression analysis of the analytical signal (peak area measured at the atomization stage) *vs*. chromium concentration and were linear in the 0.03–0.4 µg L^−1^ range (0.9983 for the regression coefficient of a typical six points calibration plot). A statistical test proved the absence of significant differences between the slopes of calibration graphs obtained from standard solutions prepared for trivalent or hexavalent chromium. The detection limit calculated on the basis of three times the standard error of the regression^38^ was found to be 0.01 µg L^−1^ chromium. The relative standard deviations for solutions containing 0.1 and 0.3 µg L^−1^ (five consecutive experiments in each case and measurements in duplicate) were 5.3 and 4.7%, respectively. It is of note that the enrichment factor, calculated as the ratio of a calibration graph divided by the slope of a calibration graph obtained from chromium solutions that were not submitted to the treatment with MNPs but directly analyzed was close to 100, which is the ratio of the sample volume used (10 mL) divided by the volume (0.1 mL) of the final solution in which the GFAAS measurement was carried out, thus confirming that chromium separation was practically total. The atomization profiles obtained when the heating program given in Table 1 was run were well-shaped, and the low background signals were easily corrected by the Zeeman device.

Table 2 summarizes the main characteristics of similar procedures reported for chromium determination at very low levels. The LOD of the procedure compares well with most of the other procedures, with the advantage of simplicity, low cost of reagents and easy preparation of the solid material. It is of note that the LOD indicated in this table (0.01 µg L^−1^ chromium) is based in a 10-mL sample aliquot. The LOD can be increased by increasing the volume of sample up to 50 mL but then reproducibility decreases. Since the limit of detection is enough low for all practical purposes the use of 10 mL for the volume of sample is recommended. The effect that the species commonly present in water samples have on the determination of chromium by application of the proposed procedure has been studied. Thus, it was experimentally verified that Na^+^, K^+^, Ca^2+^, Mg^2+^, NO_3_^−^, Cl^−^ and SO_4_^2−^ ions are tolerated up to 5 g L^−1^. Metallic ions such as Cu (II), Co (II), Ni (II), Cd (II), Al (III), Fe (III), As (III) and Sb (III) are tolerated even in a 500:1 ratio. Other metallic ions that could also be retained in the ferrite are not interfering due to the selectivity of the detection technique, provided that the retention capacity of the adsorbent material is not exceeded.

**Table 2:** Comparison of proposed procedures for the determination of Cr (VI) and/or Cr (III) using magnetic support.

Specie	Adsorbent	Reagent	Desorption	Detection	V_Sample_, mL	LOD, µg/L Cr(VI)/Cr(III)	EF, % Cr(VI)/Cr(III)	Samples	Ref.
Cr(III)	CoFe_2_O_4_	PAN	—	EDXRF	15	4	—	Etanol fuel	^39^
Cr(III), Cr(VI)	CNTM-BGs	DPC	ethanol	FO-LADS	50	0.1	318	Water	^40^
Cr(III), Cr(VI)	Fe_3_O_4_@SiO_2_@Amino	TAR	HCl 2.5 M	FAAS	45	1.1/3.2	16/12	Water and biological samples	^41^
Cr(III), Cr(VI)	Fe_3_O_4_@GO@Trien	—	NH_4_OH 2 M	FAAS	50	1.4/1.6	10	Tannery wastewater, electroplating wastewater and river water	^42^
Cr(III), Cr(VI)	Fe_3_O_4_@GO	—	HNO_3_, 0.5 M + methanol + US	FAAS	100	0.1	200	Environmental water	^43^
Cr(VI)	Fe_3_O_4_@Cr(VI)IIPS	—	HCl 1 M	FAAS	500	0.3	98	Water	^44^
Cr(VI)	Fe_3_O_4_@ADMPT	DPC	—	Vis-UV	10	2	–	Water and soils	^45^
Cr	Fe_3_O_4_@decanoic	PAN	HCl 0.25 M + propanol	FI-ICP-OES	47	0.5	120	Water	^46^
Cr(III)	Fe_3_O_4_@En/MIL 101(Fe)	—	HNO_3_ + EDTA	FAAS	1000	0.5	238	SRM and agricultural samples	^47^
Cr(III)	Fe_3_O_4_@ZrO_2_	—	HNO_3_, 0.5 M	FAAS	75	0.7	25	Environmental and biological samples	^48^
Cr(III), Cr(VI)	Fe_3_O_4_@Al_2_O_3_@Triton X-114	PAN	HNO_3_, 0.5 M	FAAS	200	1.4	120	Waters and soils	^49^
Cr(III), Cr(VI)	Fe_3_O_4_@MnO_2_,Al_2_O_3_@AAPTMS	—	HNO_3_, 2 M	ICP-OES	—	0.02	94	SRM and river waters	^32^
Cr(III), Cr(VI)	Fe_3_O_4_@En/MIL 101(Fe) /PAEDTC		HNO_3_ + EDTA	ETAAS	400	0.001	470	Water and tea	^33^
Cr(III), Cr(VI)	Fe_3_O_4_@GO@Im	—	HCl 2.2 M	ETAAS	500	1.2/1.9	357	Water	^50^
Cr(III)	Fe_3_O_4_@SiO_2_@MPA		HNO_3_, 1 M	FAAS	200	0.19	92	Biological and environmental samples	^51^
Cr(III), Cr(VI)	Fe_3_O_4_@SiO_2_@Zincon	—	HCl 2 M	ETAAS	100	0.016/0.011	100/150	Water	^52^
Cr(III),Cr(VI)	Fe_3_O_4_	—	(1)	ETAAS	10	0.01	100	Water	[*]

PAN: 1-(2-pyridylazo)-naphthol; EDXRF: energy dispersive X-ray fluorescence spectrometry; CNTM-BGs: carbon nanotube-based magnetic bucky gels; FO-LADS: fibre optic linear array detection spectrophotometer; DPC: 1,5-diphenylcarbazide; TAR: 4-(2-thiazolylazo)resorcinol; FAAS: flame atomic absorption spectrometry; Trien: triethylenetetramine; US: ultrasounds; Fe_3_O_4_@Cr(VI)IIPS: magnetic Cr (VI)-imprinted nanoparticles; ADMPT: 3-aminopropyltriethoxysilan-2,4-bis(3,5-dimethylpyrazol)triazine; FI-ICP-OES: flow injection inductively coupled plasma-optical emission spectrometry; Fe_3_O_4_@En/MIL 101(Fe): magnetic metal-organic framework nanocomposite; SRM: standard reference material; AAPTMS: [3-(2-aminoethylamino)propyl] trimethoxysilane; PAEDTC: 2-(propylamino-ethyl) dithiocarbamate; Im: imidazolium; MPA: 3-mercaptopropionic acid; (1): slurry in water; [*]: this work.

### Results for water samples and certified reference materials

The optimized procedure was used to analyze six different water samples. All of them gave signals below the detection limit with the exception of a bottled mineral water sample that contained 0.1 µg L^−1^ total chromium (0.04 µgL^−1^ for the hexavalent species), a very low level without toxicological relevance. Table 3 shows details of the recovery tests used to confirm the results.

**Table 3:** Analytical results obtained in the determination of Cr (III) and Cr (VI) in water samples.

Sample	Added, ng/L		Found, ng/L			Recovery, %	
	Cr(III)	Cr(VI)	Cr(III)	Cr(VI)	Cr (total)	Cr(III)	Cr(VI)
Tap water	0 50 100	0 50 100	<LOD 53 ± 4 109 ± 5	<LOD 47 ± 5 92 ± 5	<LOD 100 ± 5 201 ± 5	— 106 109	— 94 92
Spring water	0 50 100	0 50 100	<LOD 51 ± 4 99 ± 5	<LOD 48 ± 4 98 ± 5	<LOD 99 ± 5 197 ± 7	— 102 99	— 96 98
Sea water	0 50 100	0 50 100	<LOD 52 ± 4 106 ± 5	<LOD 47 ± 5 93 ± 5	<LOD 99 ± 5 199 ± 6	— 104 106	— 94 93
River water	0 50 100	0 50 100	<LOD 47 ± 4 107 ± 6	<LOD 54 ± 5 94 ± 5	<LOD 101 ± 5 201 ± 6	— 94 107	— 108 94
Bottled water 1	0 50 100	0 50 100	<LOD 57 ± 4 105 ± 5	<LOD 52 ± 5 92 ± 5	<LOD 109 ± 5 197 ± 6	— 114 105	— 104 92
Bottled water 2	0 50 100	0 50 100	60 ± 4 108 ± 5 158 ± 6	35 ± 4 84 ± 5 132 ± 7	95 ± 4 187 ± 6 29 ± 7	— 96 92	— 98 97

^a^Mean value of three determinations ± standard deviation.

The reliability of the results was checked by analyzing five standard reference materials with certified total chromium contents. It should be noted that, due to the sensitivity of the approach here presented, to obtain signals within the linear response range, four of these samples had to be diluted before analysis. The results given for total chromium and its speciation are given in Table 4.

**Table 4:** Analytical results obtained in the determination of Cr (III) and Cr (VI) in reference materials.

Sample	Dilution	Certified	Cr found^a^, µg L^−1^		
		Total, µg/L	Cr(III)	Cr(VI)	Cr (total)
SRM 1640a^b^	1:500	40.22 ± 0.28	16.2 ± 0.1	26.6 ± 0.2	42.8 ± 0.1
SRM TM-23.4^c^	1:50	6.77 ± 0,63	6.11 ± 0.03	0.07 ± 0,01	6.28 ± 0.09
SRM TM-25.4^d^	1:100	24.0 ± 1.73	23.2 ± 0.1	0.09 ± 0.01	23.3 ± 0.1
NASS-6^e^	—	0.116 ± 0.008	0.05 ± 0.01	0.05 ± 0.01	0.114 ± 0.003
TMRain-04^f^	1:4	0.866 ± 0.165	0.90 ± 0.05	0.02 ± 0.01	0.92 ± 0.05

^a^Mean value of three determinations ± standard deviation.

^b^Trace elements in natural water (an acidified spring water; details can be found in https://www-s.nist.gov/srmors/certificates/1640a.pdf).

^c^Fortified (high level) and acidified Lake Ontario water; details can be found in https://topslide.net/document/certified-reference-material-tm-23-4-a-trace-element-fortified-sample.

^d^Fortified (low level) and acidified Lake Ontario water.

^e^Acidified seawater (details can be found in https://nrc.canada.ca/en/certifications-evaluations-standards/certified-reference-materials/list/113/pdf/nass-6-en.pdf).

^f^Simulated rain sample for trace elements (details can be found in https://nwql.usgs.gov/Public/Performance/ECPT0098TE.pdf).

## Conclusions

The determination of chromium at low concentrations in waters can be carried out by using graphite furnace atomic absorption spectrometry, GFAAS, an analytical technique available in most laboratories and sometimes underused, despite its advantages in terms of cost and maintenance compared with inductively coupled plasma mass spectrometry, ICP-MS. The combination of a modern microextraction process with the characteristics (sensitivity and selectivity) inherent in GFAAS provides a procedure involving low cost reagents, which makes such determinations feasible in laboratories with moderate budgets. In addition to the low cost and easy availability of the reagents used, the strength of the procedure lies in its simplicity, since the synthesis of the sorbent is quite simple, not requiring any immobilization of extractive groups on its surface. The approach allows the reliable non-chromatographic speciation of chromium, even at the low concentrations usually present in waters.
